# Supplementary material for: A Likelihood Approach for Real-Time Calibration of Stochastic Compartmental Epidemic Models
Source: PLoS Comput Biol. 2017 Jan 17;13(1):e1005257. doi: 10.1371/journal.pcbi.1005257 (PMC5240920; doi:10.1371/journal.pcbi.1005257)
Supplement: S1 File — (TAR.GZ) [file pcbi.1005257.s014.tar.gz › HSPH_Online-SI-Revision/EnKF07/n100-mild/n100-mild_EnKF2_table-4.pdf]

1	newly 3 weeks spec	3503.	1805.18	{33., 1559.}	99.3194	1.10995	newly 3 weeks cumul	8908.	10 677.3	{321., 10 379.}	89.3709	0.475069	newly 5 weeks spec	3621.	699.71	{0., 987.}	92.2383	1.9565	newly 5 weeks cumul	16 248.	12 483.1	{338., 11 059.}	88.8698	0.649363	newly 56 weeks cumul	29 432.	14 081.2	{338., 11 198.}	88.8939	0.848918
2	newly 3 weeks spec	4175.	937.48	{0., 1122.}	97.6867	2.60671	newly 3 weeks cumul	10 673.	11 454.3	{927., 13 710.}	78.5146	0.397743	newly 5 weeks spec	4247.	338.27	{0., 522.}	95.9287	2.91435	newly 5 weeks cumul	19 311.	12 309.6	{1241., 13 710.}	77.5588	0.546353	newly 55 weeks cumul	34 724.	13 704.6	{2659., 30 885.}	78.0293	0.690828
3	newly 3 weeks spec	4167.	821.37	{46., 1018.}	84.5148	1.07446	newly 3 weeks cumul	10 718.	5772.09	{424., 5557.}	82.5207	0.618867	newly 5 weeks spec	4114.	356.87	{2., 582.}	93.3294	1.7505	newly 5 weeks cumul	19 213.	6649.4	{514., 5809.}	82.8931	0.773216	newly 58 weeks cumul	31 221.	7743.15	{609., 8032.}	83.8129	0.903817
4	newly 3 weeks spec	8072.	1420.73	{239., 3539.}	82.6656	1.01574	newly 3 weeks cumul	18 303.	25 112.9	{8702., 67 388.}	93.9965	0.324685	newly 5 weeks spec	6729.	298.62	{4., 325.}	95.5622	2.17914	newly 5 weeks cumul	33 292.	25 994.3	{8843., 67 682.}	69.7496	0.418794	newly 62 weeks cumul	45 410.	26 398.2	{8850., 67 709.}	65.5678	0.488386
5	newly 3 weeks spec	3934.	806.59	{20., 933.}	84.6876	1.11756	newly 3 weeks cumul	10 428.	5698.26	{438., 5693.}	83.0188	0.690577	newly 5 weeks spec	3936.	344.57	{1., 571.}	93.2203	1.79565	newly 5 weeks cumul	18 434.	6551.45	{566., 5785.}	83.0231	0.837818	newly 58 weeks cumul	32 210.	7623.54	{645., 7911.}	83.9318	0.994235
6	newly 3 weeks spec	4474.	2582.53	{20., 5646.}	105.773	1.18134	newly 3 weeks cumul	11 885.	13 842.3	{428., 24 660.}	117.036	0.655831	newly 5 weeks spec	4394.	742.85	{0., 1723.}	88.3712	1.99137	newly 5 weeks cumul	20 970.	15 945.8	{441., 31 934.}	103.98	0.811772	newly 56 weeks cumul	33 539.	17 731.3	{441., 65 766.}	94.3503	0.935101
7	newly 3 weeks spec	5537.	498.09	{0., 532.}	96.1996	2.20926	newly 3 weeks cumul	11 792.	8399.6	{971., 17 915.}	82.954	0.551562	newly 5 weeks spec	6137.	156.46	{0., 99.}	97.4505	3.15077	newly 5 weeks cumul	24 548.	8829.73	{1082., 18 034.}	82.3329	0.790013	newly 61 weeks cumul	35 993.	9093.37	{1092., 18 034.}	84.2341	0.92915
8	newly 3 weeks spec	3810.	914.77	{0., 1014.}	99.2816	2.60218	newly 3 weeks cumul	10 632.	10 757.3	{1173., 12 475.}	78.8722	0.407671	newly 5 weeks spec	3274.	338.82	{0., 527.}	95.2266	2.77901	newly 5 weeks cumul	17 804.	11 609.2	{1533., 13 256.}	80.1032	0.54201	newly 55 weeks cumul	26 742.	13 002.1	{1576., 13 610.}	80.3021	0.627667
9	newly 3 weeks spec	6820.	2459.15	{0., 4032.}	109.512	2.14091	newly 3 weeks cumul	16 298.	8169.54	{881., 27 219.}	94.5722	0.882381	newly 5 weeks spec	6191.	982.78	{0., 375.}	94.3159	2.76162	newly 5 weeks cumul	29 431.	10 992.	{881., 29 606.}	92.5924	1.06063	newly 60 weeks cumul	43 358.	12 102.3	{881., 51 011.}	89.3876	1.18583
10	newly 3 weeks spec	3895.	694.86	{4., 665.}	94.8498	1.50259	newly 3 weeks cumul	10 575.	9090.09	{1836., 6208.}	117.529	0.586182	newly 5 weeks spec	3765.	88.18	{0., 157.}	97.6579	2.43124	newly 5 weeks cumul	18 373.	9418.17	{1836., 6876.}	104.669	0.774395	newly 54 weeks cumul	31 484.	9486.22	{1836., 7182.}	97.5696	0.973873
11	newly 3 weeks spec	5349.	783.58	{17., 1028.}	86.0651	1.31413	newly 3 weeks cumul	11 903.	5636.29	{113., 5364.}	83.4636	0.78006	newly 5 weeks spec	6127.	334.94	{1., 586.}	95.0974	2.06634	newly 5 weeks cumul	24 206.	6469.49	{145., 6075.}	83.7828	0.983015	newly 58 weeks cumul	41 009.	7504.74	{173., 6894.}	85.4958	1.1355
12	newly 3 weeks spec	4227.	945.97	{1., 865.}	97.9463	1.66356	newly 3 weeks cumul	11 278.	9584.99	{1363., 8724.}	117.852	0.623983	newly 5 weeks spec	4123.	222.72	{0., 231.}	98.6165	2.48422	newly 5 weeks cumul	19 649.	10 263.6	{1376., 8818.}	104.422	0.802724	newly 54 weeks cumul	37 494.	10 469.4	{1376., 8819.}	95.8049	1.03695
13	newly 3 weeks spec	5374.	1325.38	{120., 2237.}	96.233	1.34102	newly 3 weeks cumul	12 699.	6840.88	{1292., 9332.}	102.612	0.844193	newly 5 weeks spec	5146.	353.71	{7., 638.}	94.4611	1.95616	newly 5 weeks cumul	23 362.	7862.19	{1439., 10 600.}	95.3865	1.02568	newly 59 weeks cumul	36 672.	8560.76	{1487., 13 366.}	91.5822	1.17186
14	newly 3 weeks spec	7541.	231.28	{0., 541.}	96.933	2.20873	newly 3 weeks cumul	19 010.	6889.69	{1219., 17 456.}	72.3517	0.662339	newly 5 weeks spec	5669.	44.44	{0., 85.}	99.2161	3.08869	newly 5 weeks cumul	32 236.	7028.68	{1275., 17 456.}	81.6231	0.866646	newly 61 weeks cumul	43 111.	7088.75	{1333., 17 457.}	85.1104	0.983034
15	newly 3 weeks spec	6533.	995.05	{53., 1367.}	87.5171	1.24886	newly 3 weeks cumul	14 286.	6596.67	{401., 9071.}	84.7486	0.708122	newly 5 weeks spec	6860.	461.59	{2., 598.}	93.6389	1.9644	newly 5 weeks cumul	28 288.	7718.39	{512., 14 659.}	83.8146	0.895743	newly 58 weeks cumul	44 155.	9050.76	{590., 16 005.}	85.2972	1.0178
16	newly 3 weeks spec	2942.	685.8	{1., 632.}	97.7315	1.58723	newly 3 weeks cumul	7823.	8713.23	{971., 6097.}	131.71	0.563305	newly 5 weeks spec	2786.	86.19	{0., 190.}	96.9063	2.43177	newly 5 weeks cumul	13 642.	9028.34	{971., 6300.}	111.897	0.748165	newly 54 weeks cumul	27 181.	9091.91	{971., 6300.}	99.6911	1.00441
17	newly 3 weeks spec	6206.	2885.71	{0., 5962.}	116.293	2.12	newly 3 weeks cumul	13 556.	8855.31	{906., 45 106.}	103.522	0.86125	newly 5 weeks spec	6327.	1089.65	{0., 8868.}	93.6373	2.76094	newly 5 weeks cumul	26 549.	12 096.4	{917., 60 192.}	96.5072	1.05319	newly 60 weeks cumul	43 173.	13 014.9	{917., 68 460.}	89.5314	1.20155
18	newly 3 weeks spec	7370.	7443.59	{835., 20 782.}	78.1697	0.460644	newly 3 weeks cumul	13 588.	46 849.9	{11 289., 95 883.}	255.873	0.453555	newly 5 weeks spec	8646.	2054.39	{61., 3839.}	78.4968	1.06802	newly 5 weeks cumul	31 571.	52 715.1	{12 748., 107 659.}	103.064	0.32212	newly 64 weeks cumul	43 150.	56 061.1	{13 233., 111 707.}	76.146	0.302161
19	newly 3 weeks spec	4209.	724.73	{0., 1087.}	97.1136	2.75242	newly 3 weeks cumul	10 513.	10 413.9	{602., 13 229.}	71.174	0.439785	newly 5 weeks spec	4445.	318.84	{0., 544.}	96.6371	3.06551	newly 5 weeks cumul	19 470.	11 183.4	{654., 13 229.}	74.8275	0.601661	newly 55 weeks cumul	33 382.	12 510.3	{662., 13 312.}	78.2206	0.744862
20	newly 3 weeks spec	5435.	6267.37	{952., 14 851.}	75.2182	0.353988	newly 3 weeks cumul	9479.	43 099.6	{12 620., 96 173.}	362.476	0.55154	newly 5 weeks spec	7800.	1562.17	{92., 3645.}	80.4122	1.01728	newly 5 weeks cumul	25 122.	47 672.8	{13 765., 108 196.}	119.314	0.328291	newly 64 weeks cumul	39 842.	50 192.	{14 071., 112 267.}	73.4065	0.298078
21	newly 3 weeks spec	6939.	6209.76	{839., 20 510.}	68.1208	0.407704	newly 3 weeks cumul	13 970.	40 485.1	{12 893., 92 631.}	195.815	0.365923	newly 5 weeks spec	7245.	1869.63	{46., 3733.}	77.8723	1.05899	newly 5 weeks cumul	29 481.	45 659.5	{14 230., 104 590.}	87.6406	0.26504	newly 64 weeks cumul	42 520.	48 837.6	{14 552., 108 766.}	64.3426	0.263728
22	newly 3 weeks spec	3601.	664.03	{2., 929.}	92.4074	1.51046	newly 3 weeks cumul	9861.	11 339.4	{1187., 8625.}	137.891	0.567571	newly 5 weeks spec	3826.	93.85	{0., 229.}	97.547	2.4101	newly 5 weeks cumul	17 518.	11 672.2	{1187., 8718.}	113.184	0.748835	newly 54 weeks cumul	36 374.	11 754.2	{1187., 8732.}	98.8983	1.01403
23	newly 3 weeks spec	5953.	1764.26	{137., 4757.}	98.0433	1.24011	newly 3 weeks cumul	13 022.	8859.35	{1317., 14 120.}	113.165	0.76095	newly 5 weeks spec	6752.	466.41	{11., 1300.}	93.5265	1.90468	newly 5 weeks cumul	26 990.	10 210.9	{1459., 27 877.}	97.9785	0.973336	newly 59 weeks cumul	42 711.	11 044.8	{1512., 42 951.}	91.7082	1.11085
24	newly 3 weeks spec	7664.	2314.73	{0., 8073.}	78.3929	1.93494	newly 3 weeks cumul	15 273.	33 727.9	{4921., 72 397.}	153.416	0.418473	newly 5 weeks spec	6606.	905.59	{0., 3032.}	90.6045	2.82697	newly 5 weeks cumul	30 012.	35 981.4	{6132., 72 397.}	68.2231	0.329152	newly 63 weeks cumul	39 935.	38 489.2	{7190., 73 330.}	58.7082	0.351815
25	newly 3 weeks spec	4080.	918.47	{0., 690.}	99.7056	1.82627	newly 3 weeks cumul	9690.	8991.91	{1043., 8612.}	125.803	0.684382	newly 5 weeks spec	4627.	208.02	{0., 207.}	98.536	2.63234	newly 5 weeks cumul	18 986.	9628.42	{1043., 8687.}	106.359	0.906508	newly 54 weeks cumul	37 102.	9833.75	{1043., 8737.}	96.5488	1.1496
26	newly 3 weeks spec	5222.	1628.43	{9., 1706.}	92.4537	1.28613	newly 3 weeks cumul	12 723.	10 881.4	{115., 10 593.}	92.232	0.620058	newly 5 weeks spec	5352.	695.05	{0., 1083.}	91.0417	2.28729	newly 5 weeks cumul	23 692.	12 615.9	{121., 11 243.}	91.0816	0.820425	newly 56 weeks cumul	38 625.	14 206.4	{121., 11 575.}	89.9874	0.983469
27	newly 3 weeks spec	4332.	959.68	{1., 751.}	99.6325	1.81769	newly 3 weeks cumul	10 828.	9220.64	{976., 8764.}	121.006	0.709104	newly 5 weeks spec	4581.	208.71	{0., 233.}	98.3958	2.67886	newly 5 weeks cumul	20 103.	9866.99	{976., 8859.}	104.993	0.912597	newly 54 weeks cumul	36 608.	10 080.7	{976., 8915.}	96.507	1.12902
28	newly 3 weeks spec	3391.	876.47	{0., 1128.}	98.5848	2.52544	newly 3 weeks cumul	9081.	11 141.2	{1304., 13 488.}	83.496	0.40389	newly 5 weeks spec	3182.	319.75	{0., 526.}	95.9173	2.83481	newly 5 weeks cumul	15 833.	11 940.8	{1833., 13 698.}	74.4916	0.500328	newly 55 weeks cumul	31 926.	13 275.7	{2794., 13 894.}	77.6429	0.691733
29	newly 3 weeks spec	3449.	1350.74	{71., 1410.}	98.7359	1.007	newly 3 weeks cumul	8407.	6046.6	{722., 5165.}	113.074	0.740867	newly 5 weeks spec	3362.	653.33	{23., 1413.}	90.3858	1.30096	newly 5 weeks cumul	15 415.	7586.15	{984., 6730.}	102.19	0.872871	newly 57 weeks cumul	26 694.	10 791.7	{1017., 36 081.}	98.0342	0.973266
30	newly 3 weeks spec	6917.	1329.69	{137., 2058.}	93.517	1.33734	newly 3 weeks cumul	15 845.	6905.46	{1300., 9698.}	98.3366	0.85166	newly 5 weeks spec	6557.	361.25	{6., 754.}	94.6318	2.00687	newly 5 weeks cumul	29 817.	7943.04	{1456., 10 218.}	92.4328	1.04221	newly 59 weeks cumul	44 183.	8666.44	{1505., 14 009.}	89.888	1.16598
31	newly 3 weeks spec	4050.	1813.92	{26., 1547.}	96.6998	1.1215	newly 3 weeks cumul	9667.	12 045.7	{261., 10 207.}	102.137	0.484995	newly 5 weeks spec	4228.	692.69	{1., 982.}	91.3834	1.95981	newly 5 weeks cumul	18 314.	13 832.9	{276., 10 775.}	95.9225	0.678669	newly 56 weeks cumul	33 046.	15 441.7	{280., 11 123.}	91.7146	0.870755
32	newly 3 weeks spec	3828.	683.88	{1., 684.}	95.2043	1.61031	newly 3 weeks cumul	10 480.	8980.23	{1217., 5649.}	119.465	0.617183	newly 5 weeks spec	3614.	92.72	{0., 201.}	97.4344	2.45064	newly 5 weeks cumul	17 929.	9312.93	{1229., 6275.}	105.479	0.795116	newly 54 weeks cumul	35 777.	9387.25	{1229., 6450.}	96.6013	1.05192
33	newly 3 weeks spec	6228.	6458.4	{951., 20 654.}	71.1503	0.370278	newly 3 weeks cumul	10 354.	41 381.5	{11 171., 92 867.}	308.185	0.51142	newly 5 weeks spec	7272.	1736.22	{104., 3646.}	79.1218	1.00464	newly 5 weeks cumul	26 028.	46 369.8	{12 808., 104 876.}	108.631	0.317437	newly 64 weeks cumul	32 752.	49 002.1	{14 073., 107 179.}	85.8645	0.295243
34	newly 3 weeks spec	7515.	2858.29	{0., 6170.}	108.661	2.19075	newly 3 weeks cumul	17 041.	8725.36	{912., 45 704.}	94.7766	0.877288	newly 5 weeks spec	6043.	1210.64	{0., 9092.}	94.5626	2.72331	newly 5 weeks cumul	30 687.	12 121.1	{923., 54 730.}	92.9485	1.05309	newly 60 weeks cumul	40 483.	13 594.1	{923., 80 877.}	90.8192	1.13754
35	newly 3 weeks spec	11 144.	6853.97	{983., 18 126.}	59.6374	0.479013	newly 3 weeks cumul	14 898.	48 436.9	{8186., 119 234.}	242.621	0.458617	newly 5 weeks spec	7436.	1819.	{83., 3888.}	77.6931	0.966314	newly 5 weeks cumul	37 536.	53 611.2	{9512., 122 509.}	90.9968	0.35104	newly 64 weeks cumul	40 652.	56 982.2	{13 548., 122 802.}	83.9133	0.314233
36	newly 3 weeks spec	4855.	7498.81	{873., 20 734.}	104.605	0.409509	newly 3 weeks cumul	9201.	48 880.2	{12 553., 100 301.}	436.271	0.608042	newly 5 weeks spec	5772.	1925.76	{49., 3700.}	72.9404	0.915823	newly 5 weeks cumul	20 947.	54 504.3	{12 823., 107 692.}	177.656	0.385755	newly 64 weeks cumul	32 008.	57 478.1	{13 615., 111 737.}	107.615	0.312387
37	newly 3 weeks spec	5673.	1284.94	{217., 1092.}	92.4227	1.0914	newly 3 weeks cumul	13 815.	6087.34	{1068., 4326.}	100.454	0.821068	newly 5 weeks spec	5464.	550.08	{78., 802.}	89.9616	1.42171	newly 5 weeks cumul	25 233.	7421.22	{1433., 5682.}	94.0566	0.954242	newly 57 weeks cumul	39 323.	10 575.2	{1646., 11 706.}	90.3884	1.01383
38	newly 3 weeks spec	6180.	6863.55	{1058., 19 183.}	70.6316	0.334307	newly 3 weeks cumul	10 916.	44 158.3	{12 005., 117 871.}	310.038	0.504374	newly 5 weeks spec	7800.	1799.11	{101., 3694.}	80.6068	0.968056	newly 5 weeks cumul	26 749.	49 347.2	{13 553., 121 475.}	111.434	0.311202	newly 64 weeks cumul	37 595.	52 126.1	{14 104., 121 652.}	75.7894	0.274498
39	newly 3 weeks spec	5821.	265.62	{0., 529.}	95.4369	2.10302	newly 3 weeks cumul	14 912.	6827.85	{1084., 17 572.}	70.0022	0.594958	newly 5 weeks spec	4560.	68.92	{0., 86.}	98.4886	2.99693	newly 5 weeks cumul	25 319.	7023.58	{1172., 17 585.}	77.6706	0.783834	newly 61 weeks cumul	34 866.	7160.74	{1188., 17 585.}	82.2962	0.911142
40	newly 3 weeks spec	8986.	2195.96	{0., 7283.}	81.8947	1.94441	newly 3 weeks cumul	18 610.	34 072.3	{4893., 73 044.}	118.175	0.377199	newly 5 weeks spec	6981.	815.64	{0., 2901.}	90.2601	2.91915	newly 5 weeks cumul	34 664.	36 152.4	{6037., 73 044.}	60.0087	0.331078	newly 63 weeks cumul	43 420.	38 188.7	{7190., 73 044.}	55.2146	0.355306
41	newly 3 weeks spec	8035.	7400.84	{837., 20 879.}	75.6301	0.486256	newly 3 weeks cumul	14 755.	47 076.4	{11 136., 96 050.}	231.449	0.433188	newly 5 weeks spec	8819.	2010.97	{50., 3809.}	79.1377	1.09725	newly 5 weeks cumul	33 711.	52 836.7	{12 658., 107 617.}	95.2083	0.316157	newly 64 weeks cumul	47 457.	56 068.	{13 463., 111 555.}	70.2979	0.306926
42	newly 3 weeks spec	6821.	360.6	{0., 525.}	96.6087	2.29964	newly 3 weeks cumul	14 678.	6689.84	{967., 14 883.}	75.9533	0.648708	newly 5 weeks spec	6589.	102.46	{0., 98.}	98.445	3.15006	newly 5 weeks cumul	28 856.	6976.66	{1053., 14 891.}	82.6127	0.89365	newly 61 weeks cumul	41 167.	7149.62	{1093., 14 891.}	85.7234	1.03079
43	newly 3 weeks spec	6531.	7404.73	{817., 20 674.}	83.862	0.445844	newly 3 weeks cumul	12 527.	46 927.2	{11 274., 95 964.}	284.514	0.481433	newly 5 weeks spec	8332.	2020.05	{64., 3727.}	78.4055	1.06344	newly 5 weeks cumul	29 200.	52 716.6	{12 629., 107 622.}	113.409	0.331599	newly 64 weeks cumul	45 957.	55 976.7	{13 182., 111 651.}	72.0491	0.306667
44	newly 3 weeks spec	8080.	2458.41	{1., 8195.}	76.8271	1.69965	newly 3 weeks cumul	15 523.	34 143.5	{4925., 74 812.}	154.529	0.42151	newly 5 weeks spec	8641.	904.09	{0., 3013.}	91.4275	2.80294	newly 5 weeks cumul	34 157.	36 415.1	{6064., 74 812.}	64.5441	0.347254	newly 63 weeks cumul	46 278.	38 961.6	{7059., 78 817.}	58.9083	0.389776
45	newly 3 weeks spec	4973.	1724.64	{125., 4051.}	101.653	1.27082	newly 3 weeks cumul	11 874.	8677.64	{1279., 13 747.}	116.836	0.827036	newly 5 weeks spec	4999.	444.6	{8., 1185.}	93.4499	1.87726	newly 5 weeks cumul	22 191.	9984.01	{1430., 26 740.}	102.42	1.00639	newly 59 weeks cumul	37 756.	10 801.5	{1483., 37 982.}	93.6148	1.17028
46	newly 3 weeks spec	6137.	3285.74	{0., 18 591.}	118.919	1.98704	newly 3 weeks cumul	13 041.	10 547.8	{934., 45 971.}	114.78	0.821251	newly 5 weeks spec	7235.	1248.47	{0., 8939.}	89.8901	2.67717	newly 5 weeks cumul	27 607.	14 197.4	{945., 75 089.}	98.6284	1.01889	newly 60 weeks cumul	46 416.	15 339.7	{945., 81 234.}	88.2788	1.16564
47	newly 3 weeks spec	7497.	2157.74	{0., 1700.}	106.245	2.18723	newly 3 weeks cumul	15 877.	7703.56	{907., 27 007.}	94.8002	0.877065	newly 5 weeks spec	7293.	802.27	{0., 286.}	93.6173	2.84892	newly 5 weeks cumul	31 506.	10 078.2	{913., 27 752.}	91.9171	1.09204	newly 60 weeks cumul	45 078.	10 798.	{913., 27 822.}	89.4942	1.20886
48	newly 3 weeks spec	5435.	716.74	{0., 1164.}	95.756	2.8377	newly 3 weeks cumul	12 559.	10 236.8	{730., 12 455.}	69.4362	0.481913	newly 5 weeks spec	5927.	317.69	{0., 544.}	96.9976	3.15538	newly 5 weeks cumul	24 449.	10 997.	{1106., 12 485.}	77.4635	0.68592	newly 55 weeks cumul	39 759.	12 342.3	{2242., 13 410.}	80.05	0.811152
49	newly 3 weeks spec	5594.	380.35	{0., 539.}	95.8	2.19107	newly 3 weeks cumul	12 132.	7640.84	{1021., 14 483.}	80.3924	0.563263	newly 5 weeks spec	4978.	111.13	{0., 92.}	97.7676	3.01336	newly 5 weeks cumul	22 959.	7950.15	{1109., 14 494.}	82.4022	0.779553	newly 61 weeks cumul	32 589.	8161.63	{1122., 14 494.}	84.3912	0.907819
50	newly 3 weeks spec	6652.	1405.23	{234., 3452.}	80.8811	0.935435	newly 3 weeks cumul	14 670.	24 948.7	{7159., 67 477.}	108.857	0.300991	newly 5 weeks spec	6004.	274.57	{6., 319.}	95.4269	2.09959	newly 5 weeks cumul	27 698.	25 769.6	{8611., 67 898.}	74.9535	0.390452	newly 62 weeks cumul	37 321.	26 153.3	{8861., 67 909.}	67.1234	0.445244
